# NLRP3 mediates lipid-driven macrophage proliferation in established atherosclerosis

**DOI:** 10.1007/s00395-025-01137-6

**Published:** 2025-09-16

**Authors:** Carmen Härdtner, Felix Remmersmann, Carolin Ehlert, Christina Zehender, Tamara Antonela Vico, Bianca Dufner, Alexander von Ehr, Julia Hinterdobler, Xiaowei Li, Guido Pisani, Filip K. Swirski, Constantin von zur Mühlen, Dennis Wolf, Martin Czerny, Olaf Groß, Hendrik B Sager, Dirk Westermann, Ingo Hilgendorf

**Affiliations:** 1 Department of Cardiology and Angiology, University Heart Center Freiburg-Bad Krozingen, Freiburg, Germany; 2 Faculty of Medicine, University of Freiburg, 55 Hugstetter Street, 79106 Freiburg, Germany; 3 Department of Cardiology, German Heart Center Munich, Technical University Munich, Lazarettstr. 36, 80636 Munich, Germany; 4 Cardiovascular Research Institute, Cardiology, Icahn School of Medicine at Mount Sinai, New York, NY USA; 5 Institute of Neuropathology, University Medical Center, University of Freiburg, Freiburg, Germany

**Keywords:** Atherosclerosis, Macrophages, Proliferation, NLRP3, MCC950

## Abstract

An increased number of macrophages in the atherosclerotic plaque is associated with plaque instability and plaque progression. Lowering systemic cholesterol levels suppresses local macrophage proliferation and leads to plaque regression. However, the pathways regulating macrophage proliferation remain poorly understood. We investigated the cellular processes that underlie lipid-triggered local macrophage proliferation in the atherosclerotic plaque in transgenic mice and in human plaque tissue samples. Macrophages from mice with genetic deficiencies in scavenger receptors *Cd36*^*−/−*^ and *Msr1*^*−/−*^ showed reduced lipid uptake, lower intracellular lipid content, and lower proliferation compared to wild type macrophages. Double knockouts for the cholesterol exporters *Abca1* and *Abcg1* (MAC-ABC-DKO) showed increased rates of macrophage proliferation and apoptosis. In *Cd36*^*−/−*^, *Msr1*^*−/−*^, and MAC-ABC-DKO mixed bone marrow chimeras, no differences in chimerism were observed in blood or aorta after 4 weeks on a high-cholesterol diet. After 12 weeks of atherogenic diet, wild type macrophages predominated in the aorta since they proliferated more than neighboring *Cd36*^*−/−*^ or *Msr1*^*−/−*^ macrophages, and were less apoptotic than ABC-DKO macrophages, respectively. Knockout of NLRP3, but not ASC, Caspase 1 or IL-1 receptor, limited macrophage proliferation; indicating an NLRP3-dependent, but inflammasome-independent, effect. Inhibition of NLRP3 by MCC950 in human carotid artery plaque tissue cultures resulted in the suppression of intra-plaque macrophage proliferation and IL-1β release consistent with murine in vivo data. We identified a novel role for NLRP3 in driving macrophage proliferation in atherosclerotic plaques. NLRP3 inhibition may represent an ideal therapeutic target in atherosclerosis by combining anti-inflammasome and anti-proliferative effects in macrophages.

**Supplementary Information:**

The online version contains supplementary material available at 10.1007/s00395-025-01137-6.

## Introduction

Cardiovascular diseases are the leading cause of death worldwide [40], and in most cases atherosclerosis is the underlying pathological mechanism [14]. Atherosclerosis is a chronic inflammatory disease in which macrophages play a major role; increased numbers of macrophages and lipids in the atherosclerotic plaque are associated with plaque instability and plaque progression [12, 17, 21, 38]. Plaque progression is characterized by the uptake of subintimal, accumulated, modified low-density lipoprotein (LDL) through scavenger receptors on macrophages and foam cell formation [12, 24, 26].

Prior work has shown that lipids trigger local macrophage proliferation in the atherosclerotic plaque. Systemic lipid reduction, achieved through both oral administration of the lipid-lowering drug statin and the use of a lipid-free diet, leads to diminished lesional macrophage proliferation and plaque regression in mice [12]. Deletion of the scavenger receptors CD36 and macrophage scavenger receptor 1 (MSR1) on macrophages in chimeric, atherosclerotic mice causes the blockade of cholesterol-rich modified LDL uptake into plaque macrophages and also leads to reduced macrophage proliferation in the plaque [12, 31]. Nevertheless, the underlying mechanism by which lipids influence macrophage proliferation remains unknown.

In this study, we aimed to determine how lipids trigger local macrophage proliferation in the atherosclerotic plaque, to identify mediators of atheromatous plaque macrophage proliferation using in vivo mixed bone marrow chimeric mouse models, and to identify a possible therapeutic target for translational research in human atherosclerotic plaque tissue.

## Material and methods

### Animals and diet

8-week-old, female *Ldlr*^*–/–*^ mice (B6.129S7-Ldlrtm1Her/J) were lethally irradiated (10 Gy, irradiator IBL637C g-ray 137Cs; Schering Cis-Bio International, France) and reconstituted with a 1:1 mixture of 3,000,000 bone marrow cells from CD45.1 C57Bl/6 (B6.SJL-Ptprca Pepcb/BoyJ) and CD45.2 (*Msr1*^*–/–*^ [B6.Cg-Msr1tm1Csk/J], *Cd36*^*–/–*^ [B6.129S1-CD36tm1Mfe/J], *Myd88*^*–/–*^ [B6.129P2(SJL)-Myd88^tm1Defr^/J]) *Lxrα/β*^*−/−*^, *Nlrp3*^*–/–*^ (B6N.129-Nlrp3^tm2Hhf^/J), *Pycard*^*–/–*^ (B6.129-PycardtmTsc), *Casp1*^*–/–*^ (B6-Casp1tm2Gross/N), *Il-1r*^*–/–*^ (B6.129S7-Il1r1^tm1lmx^/J), LysM^Cre/WT^:ABCA1/G1^fl/fl^ (B6.129P2-Lyz2tm1(cre)Ifo/J, Mac-ABC-DKO, in-house breeding) mice, as previously described [31]. Mice were purchased from The Jackson Laboratory (Bar Harbor, ME, USA). Following 6 weeks of reconstitution consuming chow diet, mice were fed a high-cholesterol diet (HCD) (1.25% w/w cholesterol, D12108 mod.; Ssniff GmBH, Soest, Germany) for 4 or 12 weeks to study early and advanced atherosclerosis, respectively. Mice were housed under specific pathogen-free conditions.

8-week-old *Apolipoprotein E* (*Apoe*)-deficient mice were sex-matched and randomly assigned to 2 groups. They were fed a cholesterol diet (21.2% fat by weight and 0.2% cholesterol, TD.88137, Envigo) for 12 weeks. Four weeks prior to euthanasia, mice received either placebo or MCC950 (weight-adjusted 10 µg/g) by intraperitoneal injection every two days. To visualize local macrophage proliferation, 125 µg 5-ethynyl-2-deoxyuridin (EdU) (ThermoFisher Scientific, Waltham, MA, USA) was injected intraperitoneally for the last three hours prior to sacrifice.

### Human plaques

Tissue containing atherosclerotic plaques was collected from patients within 6 h after elective eversion carotid endarterectomy. All patients consented to take part in the study prior to surgery (Freiburg ethic approval no. 249/14). Residual blood and adventitial fat were removed from the specimens, and plaques were cut into 2 mm thick cross-sections. Three adjacent sections were transferred into 1 ml of medium each, with an EdU concentration of 50 µmol/L. Sections were either treated with MCC950 (1 µmol/L), with MCC950 (1 µmol/L) plus recombinant human interleukin-1 beta (IL-1β) (125 pg/ml), or with vehicle (control) for 24 h. After 24 h of treatment, the supernatants were collected and used to determine IL-1β concentrations. Tissues were embedded in Tissue-Tek OCT (Sakura Finetek, Torrance, CA, USA), frozen and cut into 10 µm slices. For staining, slices were fixed in cold acetone and permeabilized using 0.1% TritonX 100 solution (Applichem GmbH, Darmstadt, Germany); the Click-iT EdU Imaging Assay Kit (ThermoFisher Scientific, Waltham, MA, USA) was then used according to the manufacturer's protocol. For blocking, the sections were incubated with 5% goat serum and incubated with primary CD68 antibody (Clone KP1, Bio-Rad Laboratories, Hercules, CA, USA, 1:250) overnight. The secondary antibody (goat anti-mouse, 1:500; Goat anti Mouse IgG H&L AF647 preadsorbed; Abcam, Cambridge, UK) was added to the sections for 4 h. Finally, the sections were treated with the Vector TrueVIEW Autofluorescence Quenching Kit (Vector Laboratories, Burlingame, CA, USA) to reduce autofluorescence, and were stained with 4′,6-diamidino-2-phenylindole (DAPI) (Vector Laboratories, Burlingame, CA, USA).

### Bone marrow-derived macrophages (BMDMs)

Bone marrow cells were extracted from femurs and tibiae. The cell suspension was processed through a 40 μm cell strainer. For macrophage differentiation, 10^5^ bone marrow cells were cultured in 1 ml conditioned media (RPMI with 10% fetal calf serum (FCS); 1% nonessential amino acids (NEAA); 1% penicillin/streptomycin (PenStrep); 30 ng/ml murine macrophage colony stimulating factor (M-CSF; PeproTech, Hamburg, Germany)) per well in a 48-well plate for 5–7 days. The media, with supplements, was changed every two days.

### Macrophage uptake of oxidized (ox)LDL or acetylated (ac)LDL

Murine BMDM were incubated with 20 μg/ml human DiI-labeled medium oxidized LDL or Dil-labeled acetylated LDL (DiI-oxLDL/ Dil-acLDL, Kalen Biomedical, Montgomery Village, MD, USA) for 4 h. Foam cell formation and differentiation were analysed by flow cytometry (defined as viability dye^low^ F4/80^high^ Dil-positive macrophages and their mean fluorescent intensity, MFI).

### BODIPY-cholesterol uptake

Murine BMDMs were incubated for 90 min with 0.025 mM TopFluor Cholesterol (BODIPY-cholesterol, Avanti Polar Lipids, Alabaster, USA) in cell culture medium. The medium was removed, and the cells were incubated overnight with 2 μg/ml acyl-coenzyme A:cholesterol O-acyltransferase (ACAT) inhibitor (Tocris Bioscience, Wiesbaden, Germany) in cell culture medium with 2 g/L bovine serum albumin (BSA). Cholesterol efflux was initiated by adding 25 μg/ml human high-density lipoprotein (HDL, Kalen Biomedical, Montgomery Village, MD, USA) to the culture media for 4 h.

The supernatants were collected, centrifuged, and analyzed with a SpectraMax M2 plate reader (Molecular Devices, San Jose, CA, USA) with a 490 nm excitation filter, 520 nm emission filter, and 515 nm cutoff. The amount of cholesterol efflux was quantified using linear regression (5-point calibration on concentrations ranging from 1 to 25 μM TopFluor Cholesterol).

### BMDM proliferation assay

After differentiation, murine *NOD-like receptor family pyrin domain containing 3* knockout (*Nlrp3*^*−/−*^) and wild-type (WT) BMDMs were incubated for 12 h in cell culture medium with 20 µg/ml human Dil-labeled oxLDL (DiI-oxLDL; Kalen Biomedical, Montgomery Village, MD, USA). In order to compare proliferation between the two different BMDM populations, 10 µmol/ml EdU was added to each well and its incorporation in proliferating cells was measured by flow cytometry (defined as viability dye negative, F4/80^high^ CD11b-positive macrophages).

### RNA Sequencing

Cells were lysed, and RNA was prepared using the RNeasy Micro Kit (Qiagen, Hilden, Germany) according to the manufacturer’s protocols. An RNA library was prepared according to the NEBNext Low Input RNA Library Prep Kit for Illumina (New England Biolabs, Ipswich, MA, USA).

Paired-end sequencing was performed via the European Molecular Biology Laboratory (EMBL). Quality Control and Data Prepocessing was performed in Galaxy [38]. Tools were used with default settings unless stated otherwise. *FastQC*-reports were used to assess data quality repetitively during quality control and data preprocessing [1]. Cutadapt was used to trim adapters and low-quality bases [25]. Trimmed reads were mapped to a built-in reference genome (mm10) using RNA STAR [6].

After mapping, gene expression was measured using the featureCounts program and a built-in reference genome was used for annotation [14]. Raw read counts were acquired for differential expression analysis. Batch effects included the date of harvest and the origin of cells regarding individual test animals, and these were included in subsequent analysis. Differential expression analysis was performed in RStudio (RStudio-2023.06.1–524) using the packages DESeq2 and dplyr. Genes with a cumulative read count lower than 10 were filtered out. Benjamini–Hochberg correction was used for multiple testing corrections. Gene ontology analysis was performed in Metascape [43] and DAVID. Gene set enrichment analysis was performed in GSEA on the curated M2cp gene sets of the Molecular Signatures Database (MSigDB), including various gene sets that are expert-confirmed canonical representations of biological processes [34].

### Aortic cell isolation and flow cytometry

Murine aortic cells were retrieved by enzymatic digestion with a two-stage procedure. First, tissue was digested with collagenase I, collagenase XI, hyaluronidase, and DNAse I solution (Sigma-Aldrich, St. Louis, MO, USA) in a thermocycler for 70 min at 250 rpm and 37 °C, followed by a second digest with collagenase II (Worthington Biochemical Corp., Lakewood, NJ, USA) and hyaluronidase for 8 min at 250 rpm and 37 °C. Murine blood samples were lysed in red blood cell lysis buffer (Biolegend, San Diego, CA, USA). Isolated cells from blood and aorta were counted using a Neubauer chamber (Marienfeld, Lauda-Königshofen, Germany). Cells were stained with specific fluorescent antibodies as specified in Supplemental Table S1.

Chimerism shifts were quantified by comparing aortic monocytes and macrophages to their circulatory precursors in blood using flow cytometry. The baseline WT/KO ratio was determined from Ly6C^high^ blood monocytes (CD45.1^+^ or CD45.2^+^, CD11b^+^, Lin^−^ (where Lin indicates CD3, CD19, NK1.1, Ly6G), Ly6C^high^, CD115 + , F4/80^low^). Aortic monocytes (Ly6C^high^, (CD45.1^+^ or CD45.2^+^, CD11b^+^, F4/80^low^ or CD68^+^) and macrophages (CD45.1^+^ or CD45.2^+^, CD11b^+^, F4/80^high^ or CD68^+^) were similarly analyzed. Shifts from baseline were calculated as Δ of mean WT/KO ratio in blood monocytes minus respective WT/KO ratio in aortic monocytes or macrophages. Positive Δ indicates enrichment of the respective WT population; a negative Δ indicates enrichment of KO population.

To assess proliferation and apoptosis, intracellular staining with anti-Ki-67 and anti-active Caspase-3 (Casp3) was performed using BD Cytoxfix/Cytoperm (#554,722, BD Biosciences, San Diego, CA, USA), BD Perm/Wash (#554,723, BD Biosciences, San Diego, CA, USA) and BD Permeabilization Buffer Plus (#561,651, BD Biosciences, San Diego, CA, USA), following the manufacturer’s instructions.

The gating strategy of *Low-density lipoprotein receptor knockout* [*Ldlr*^*−/−*^] chimera is shown in Supplemental Fig. S1b. Flow cytometry analysis of *Apoe*^*−/−*^ mice was performed using the same gating strategy, as shown in Supplemental Fig. S2a.

Data were collected on a BD FACS Canto II (BD Biosciences, San Diego, CA, USA) and analyzed with FlowJo (Tree Star, Ashland, OR, USA).

### Histology

Murine aortic roots were embedded in Tissue Tek Optimal cutting temperature (OCT) compound (Sakura Finetek, Tokyo, Japan) and cut into serial 5 µm cryostat sections, starting at the level of the aortic valve. Sections were stained with anti-CD68 (clone FA-11, BioRad AbD Serotec, Puchheim, Germany), anti-CD45 (AF114, R&D Systems, Minneapolis, MN, USA), secondary antibodies Donkey α-Goat AF488 (Abcam, Cambridge, UK), rabbit α-rat AF647 (Abcam, Cambridge, UK), and Click-iT EdU Imaging Assay Kit (Thermo Fisher Scientific, Waltham, MA, USA) according to the manufacturers' instructions. Images were recorded with the Axio Imager.Z2 (Carl Zeiss Micro Imaging GmbH, Göttingen, Germany). Images were analyzed with Image Pro Premiere 9.2 (Media Cybernetics, Rockville, MD, USA) or Zeiss Zen Lite (Carl Zeiss MicroI maging GmbH, Göttingen, Germany) and manually counted. Human plaque histology methods are described in 2.2.

### Statistics

Results are presented as mean ± SEM. Differences between two groups were analyzed with paired Student’s t-test as indicated in the figure legends. To assess differences between more than two groups, one-way ANOVA with Holm–Šídák test for multiple comparisons was used. p values ≤ 0.05 indicate significant changes. Pearson’s correlation coefficient was used to test for correlation.

## Results

### Lipid uptake triggers macrophage proliferation in the atherosclerotic plaque

**Fig. 1:**
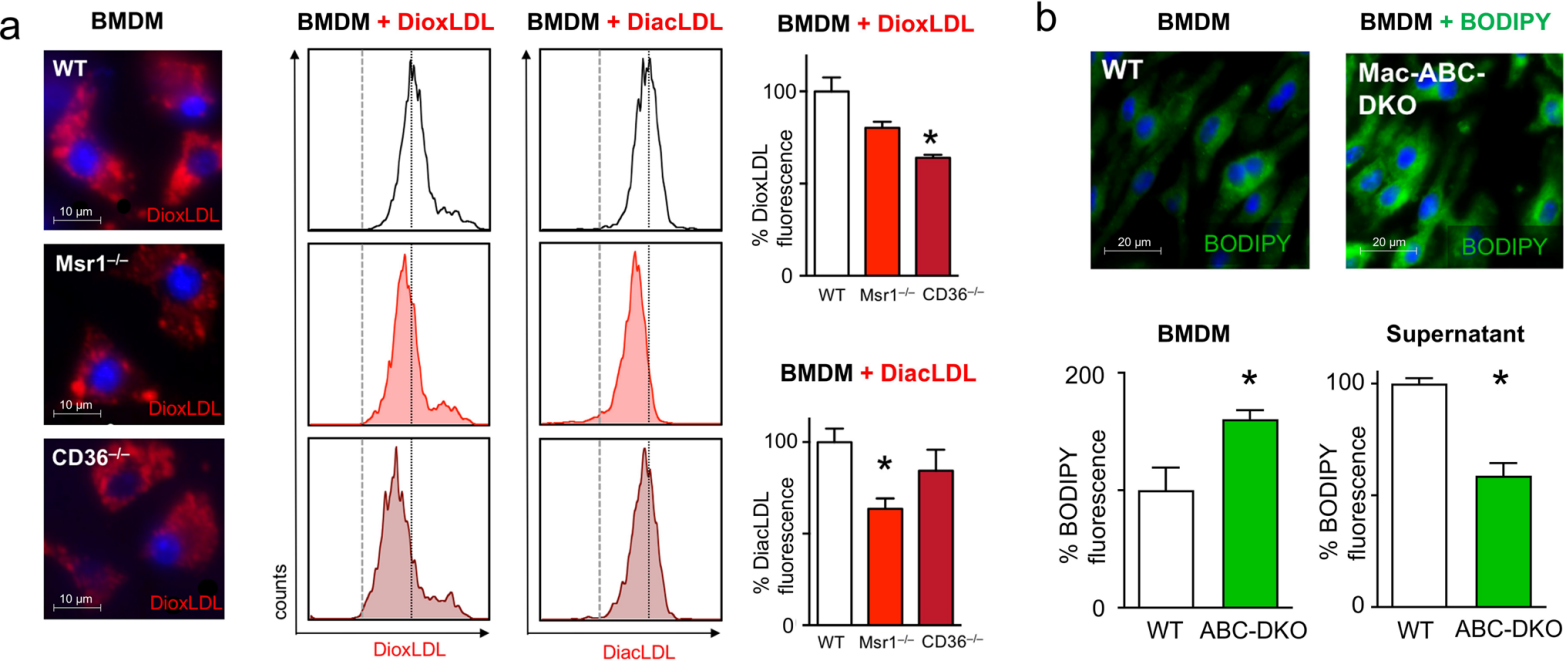
Experimental modulation of lipid uptake and export in BMDMs in vitro **a** BMDM from WT, *CD36*^*−/−*^, and *Msr1*^*−/−*^ mice were incubated with fluorescent Dil-oxLDL and Dil-acLDL, and the uptake was quantified by flow cytometry. Modified LDL uptake is shown as mean ± SEM percentage change versus WT; n = 3–4; *p < 0.05 indicates statistically significant differences as determined by one-Way ANOVA (Kruskal–Wallis test). Representative pictures of Dil-oxLDL uptake (red fluorescence) in wild type, *Msr1*^*−/−*^ and *Cd36*^*−/−*^ BMDM are shown. **b** Relative BODIPY-cholesterol accumulation is shown in Mac-*Abc*-DKO BMDM compared to WT BMDM; data are mean ± SEM percentage change; n = 5 *p < 0.05 indicates statistically significant differences as determined by t-test. Representative pictures of BODIPY-cholesterol (green fluorescence) uptake in *Abc* -DKO macrophages are shown

In order to analyze the effect of lipid uptake on macrophage proliferation, we incubated BMDM isolated from mice carrying knockouts for *Cd36* or *Mrs1* with fluorescent Dil-oxLDL or Dil-acLDL. Macrophage Dil-oxLDL and Dil-acLDL uptake was analyzed using flow cytometry (Fig. 1a).

Dil-oxLDL and Dil-acLDL uptake was lower in *Cd36* and *Msr1* deficient macrophages when compared with WT macrophages (Fig. 1a). *Cd36* knockout led to a greater reduction in Dil-oxLDL uptake (35.9% ± 7.7%) compared to Dil-acLDL (15.6% ± 11.4%), whereas *Msr1* knockout significantly limited the uptake of Dil-acLDL in macrophages to a greater extent (36.3% ± 5.5%) when compared with Dil-oxLDL (19.3% ± 3.3%) (Fig. 1a), suggesting that the cholesterol importer CD36 and MSR1 are the main receptors mediating macrophage uptake of Dil-oxLDL and Dil-acLDL, respectively (Fig. 1a).

To assess the effects of lipid export, we compared relative BODIPY-cholesterol accumulation in BMDMs derived from macrophage-specific double knockout mice lacking the cholesterol exporters ABCA1 and ABCG1 (Mac-ABC-DKO) with that in wild-type mice, using BODIPY fluorescence (Fig. 1b). *Abc*-DKO BMDM showed a 60.6% ± 7.4% increase in intracellular cholesterol and, correspondingly, decreased cholesterol concentration in the supernatant (Fig. 1b).

**Fig. 2:**
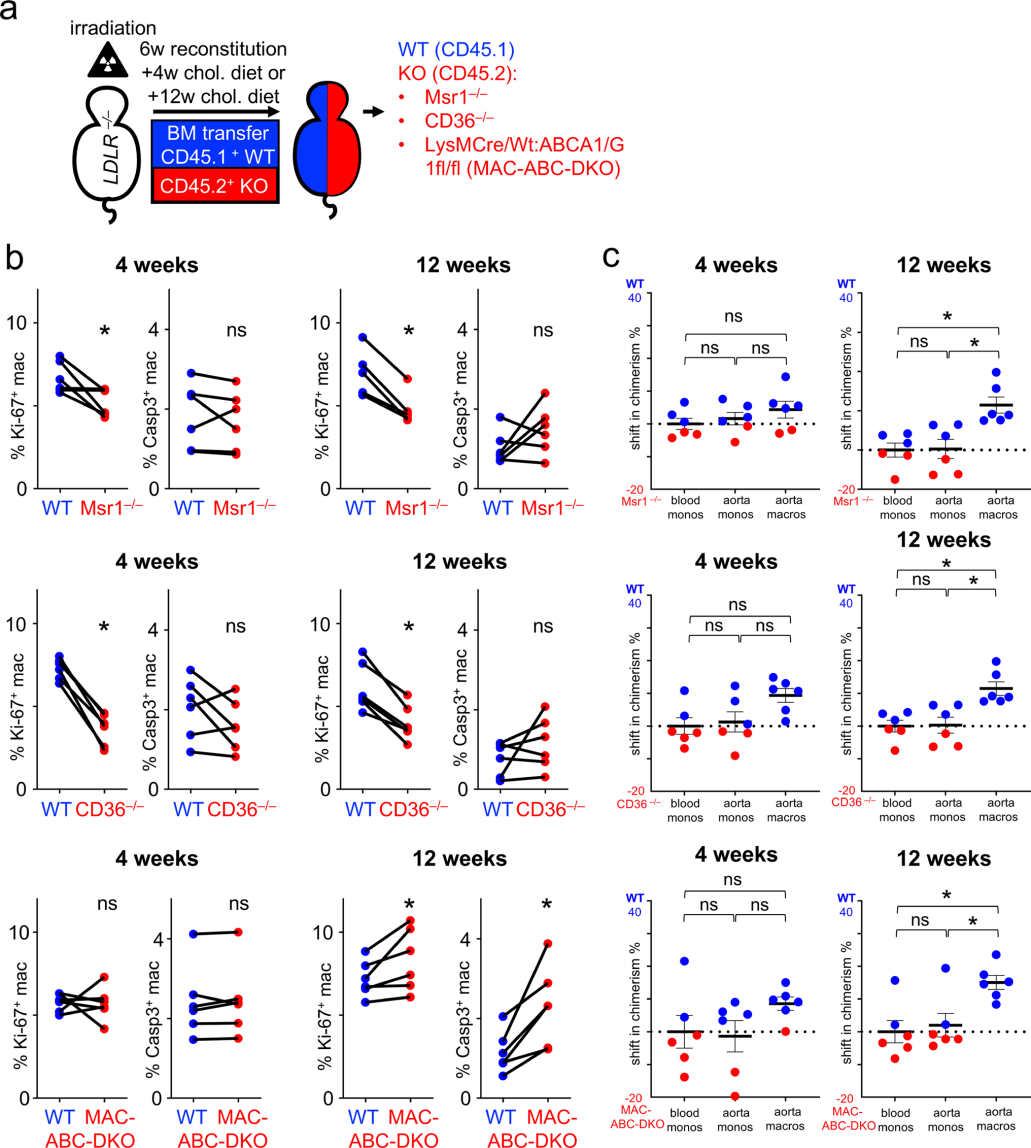
Lipids trigger macrophage proliferation in the atherosclerotic plaque **a** In the chimera model, irradiated *Ldlr*^*−/−*^ mice were reconstituted with a 1:1 mixture of CD45.1 WT and CD45.2 KO bone marrow cells as indicated. After 6 weeks of reconstitution, mice were fed a HCD for 4 or 12 weeks to study early and advanced atherosclerosis, respectively. **b** Based on intracellular Ki-67 and active Caspase-3 expression, proliferation and apoptosis of WT and KO macrophages were analyzed by flow cytometry after 4 and 12 weeks of HCD feeding. Results are presented as individual proliferation and apoptosis fraction between WT and the respective KO; n = 6; ns = not significant, *p < 0.05 indicates statistically significant differences as determined by t-test. **c** After 4 and 12 weeks of HCD feeding, the chimerism of cell populations in the blood and the digested aortas of the mice were analyzed by flow cytometry. The shift in chimerism is shown as mean ± SEM; n = 6; *p < 0.05 indicates statistically significant differences as determined by one-way ANOVA

We then generated mixed chimeric mice using *Cd36*^*−/−*^, *Msr1*^*−/−*^, Mac-*Abc*-DKO, and WT mice as bone marrow donors. *Ldlr*^*–/–*^ mice were lethally irradiated and reconstituted with a 1:1 mixture of CD45.2 gene-deficient (− / −) and CD45.1 wild type (+ / +) bone marrow cells (Fig. 2a, Suppl. Fig. S1a). Using this approach, direct comparison of CD45.1 and CD45.2 cells within the same mouse provides insight into the cell-intrinsic functions of the respective knockout gene under atherogenic conditions [12].

Six weeks after reconstitution, chimeric *Ldlr*^*−/−*^ mice were fed a high-cholesterol diet for either 4 weeks, representing early atherosclerosis, or 12 weeks, representing advanced atherosclerosis (Fig. 2a).

After 4 weeks of feeding with a high-cholesterol diet, the monocyte chimerism in blood and aorta did not differ between CD45.1 wild type and CD45.2 knockout cells in *Msr1*^*−/−*^, *Cd36*^*−/−*^, and Mac-*Abc*-DKO chimeras (Fig. 2c). In all three chimera models, wild type and knockout macrophage numbers in the plaque were consistent with the numbers of monocytes in the blood and aorta (Fig. 2c). In advanced atherosclerosis (i.e. after 12 weeks of feeding with a high-cholesterol diet), wild type macrophages predominated over knockout macrophages in the aortas of *Msr1*^*−/−*^, *Cd36*^*−/−*^, and Mac-*Abc*-DKO chimeras, but no difference was seen in blood or aortic monocytes (Fig. 2c). Monocyte infiltration and differentiation, which may have otherwise contributed to altered macrophage counts in the plaque, were unaffected in the chimera mice, suggesting a local effect on macrophage turnover.

We next analyzed macrophage proliferation, as assessed by Ki-67 staining, and cell death, assessed by active Caspase-3 staining, by flow cytometry (Fig. 2b). CD45.1 wild type aortic macrophages showed greater proliferation (6.68% [± 0.39%] and 7.29% [± 0.26%], respectively) than the corresponding CD45.2 *Msr1*^*−/−*^ or *Cd36*^*−/−*^ macrophages (5.21% ± 0.33% or 3.63% ± 0.39%, respectively) (Fig. 2b). This effect was already significant after just 4 weeks of feeding with a high-cholesterol diet but was also observed after 12 weeks of high-cholesterol diet (Fig. 2b). Cell death remained unaffected in *Cd36*^*−/−*^ and *Msr1*^*−/−*^ aortic macrophages (Fig. 2b). Differences in Ki-67 expression between CD45.1 WT and CD45.2 *Msr1*^*−/−*^ or *Cd36*^*−/−*^ macrophages reliably reflect the effects of the respective knockouts (KO), as CD45.2 WT macrophages exhibit comparable proliferation rates to CD45.1 WT macrophages in control chimeras (Suppl. Fig. S1b).

The anti-proliferative effect of CD45.2 *Msr1*^*−/−*^ and *Cd36*^*−/−*^ macrophages versus CD45.1 WT macrophages appeared to increase during disease progression; furthermore, whereas changes in chimerism were observed in aortic macrophages in late atherosclerosis, no alteration in chimerism in aortic macrophages was seen in early atherosclerosis. This effect was even more distinct in *Cd36*^*−/−*^ chimera when compared with *Msr1*^*−/−*^ chimera, with a 32.4 ± 1.41% change in chimerism towards wild type for *Cd36*^*−/−*^ chimeras, versus a 11.45 ± 2.07% change for *Msr1*^*−/−*^ chimera (Fig. 2b).

In Mac-*Abc*-DKO chimeras, wild type aortic plaque macrophages showed a slightly lower degree of proliferation versus knockouts after 12 weeks on high-cholesterol diet (7.19 ± 0.44% and 8.34 ± 0.77% Ki-67 for Mac-*Abc*-DKO and wild type, respectively). The predominant effect in these chimeras, however, appeared to be cell death; among lesional CD45.2 Mac-*Abc*-DKO macrophages, the rate of apoptosis was increased by about 50% compared to the corresponding CD45.1 WT macrophages after 12 weeks on high-cholesterol diet (1.16 ± 0.21% and 2.32 ± 0.41% active Caspase-3 for WT and Mac-*Abc*-DKO) (Fig. 2b). This effect is reversed and attenuated in mice fed a high-cholesterol diet for 4 weeks (Fig. 2b). The end result appears to be a net effect wherein CD45.1 WT aortic macrophages slightly predominate over CD45.2 Mac-*Abc*-DKO macrophages in advanced lesions (with a 15.06 ± 2.15% shift towards wild type) (Fig. 2c).

### Intracellular cholesterol sensing via Liver X receptor alpha and beta or toll-like receptor co-signaling are dispensable for plaque macrophage proliferation

**Fig. 3:**
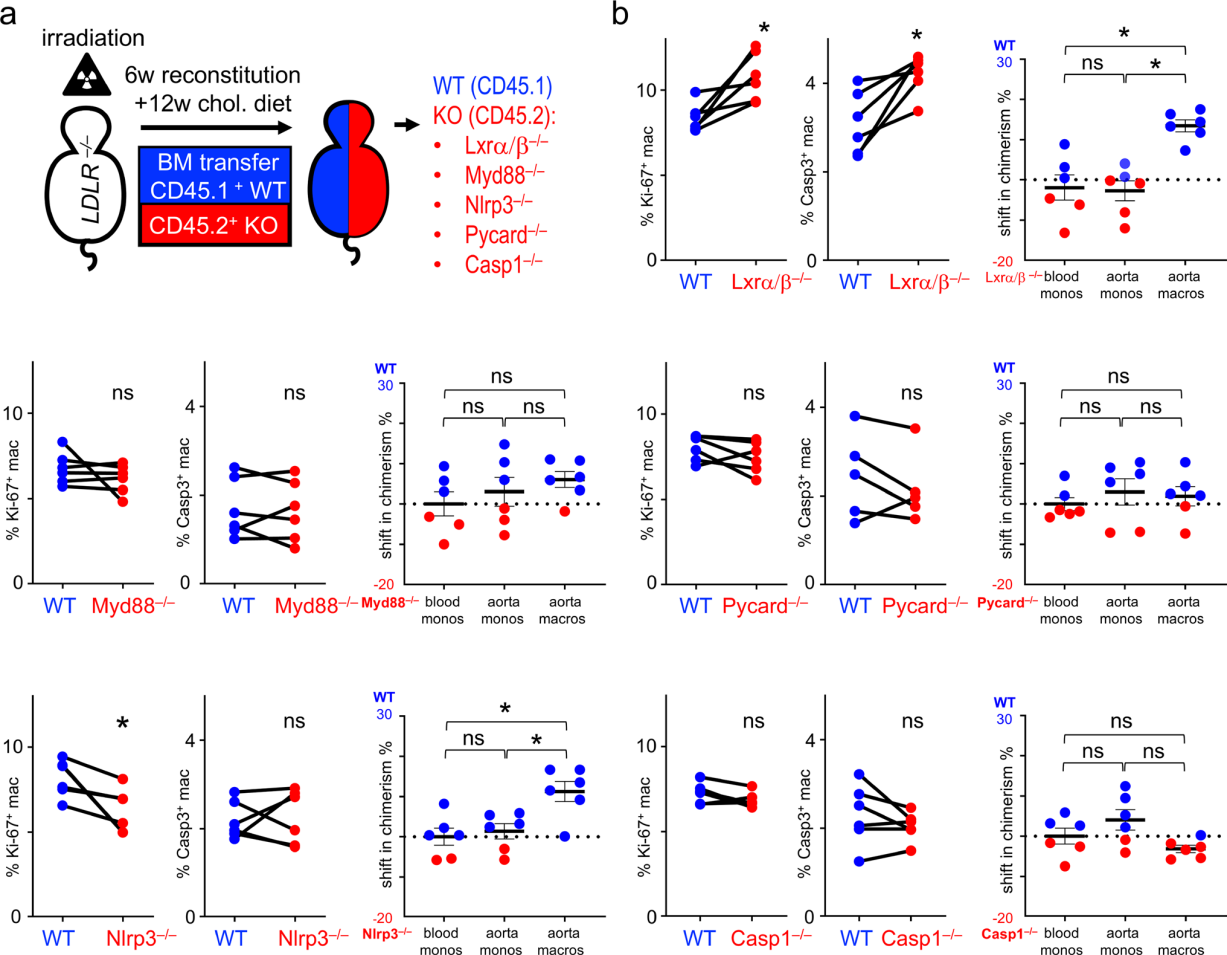
NLRP3 mediates macrophage proliferation in established atherosclerosis **a** In the chimera model, irradiated *Ldlr*^*−/−*^ mice were reconstituted with a 1:1 mixture of CD45.1 WT and CD45.2 KO bone marrow cells as indicated. After 6 weeks of reconstitution, mice were fed a HCD for 12 weeks. **b** After 12 weeks of HCD feeding, the chimerism of cell populations in the blood and the digested aortas of the mice were analyzed by flow cytometry. The shift in chimerism is shown as mean ± SEM; n = 6; * p < 0.05 indicates statistically significant differences as determined by one-Way ANOVA. Based on intracellular Ki-67 and active Caspase-3 expression, proliferation and apoptosis of WT and KO macrophages were analyzed by flow cytometry. Results are presented as individual proliferation and apoptosis fraction between WT and the respective KO; n = 6; ns = not significant, *p < 0.05 indicates statistically significant differences as determined by t-test

Next, we aimed at studying potential intracellular pathways that mediate lipid uptake-associated macrophage proliferation in the atherosclerotic plaque (Fig. 3a).

We first generated mixed *Nr1h3/2* (*Lxrα/β*) knockout chimeras. LXRα/β acts as a sterol sensor and is being activated upon increased intracellular cholesterol concentrations [27]. After 12 weeks on a high-cholesterol diet, wild-type macrophages accumulated more prominently than *Lxrα/β*^*−/−*^ macrophage in atherosclerotic plaques of mixed chimeric mice (13.82 ± 1.49% change in chimerism towards wild type) (Fig. 3b). Resembling the Mac-ABC-DKO phenotype, proliferation and apoptosis were relatively increased in *Lxrα/β*^*–/–*^ macrophages (proliferation was 8.37 ± 0.35% and 10.81 ± 0.58% for WT and *Lxrα/β*^*−/−*^ macrophages, respectively; apoptosis was 3.1 ± 0.29% and 4.21 ± 0.21% for WT and *Lxrα/β*^*−/−*^ macrophages, respectively) (Fig. 3b). Oxidized lipoproteins can activate toll-like receptors 2 and 4 [4], both of which signal via the adaptor protein MYD88. After transplantation of CD45.2 *Myd88*^*−/−*^ bone marrow mixed with CD45.1 WT bone marrow into irradiated *Ldlr*^*–/–*^ mice and 12 weeks on a high-cholesterol diet, knockout macrophages isolated from atherosclerotic aortas showed no significant differences in chimerism, proliferation, or apoptosis compared to the corresponding wild type cells (Fig. 3b). In summary, neither LXR nor MYD88 appeared to mediate lipid-associated plaque macrophage proliferation in vivo.

### NLRP3 mediates macrophage accumulation and proliferation

Next, we analyzed components of the NLRP3 inflammasome pathway, specifically NOD-, LRR- and pyrin domain-containing protein 3 (NLRP3), Apoptosis-associated speck-like protein containing a CARD (ASC), Caspase 1, and interleukin 1 receptor (IL1-R). Macrophages with deletion of Pycard coding for ASC or Casp1, coding for Caspase 1, showed no significant differences in chimerism, proliferation, or apoptosis compared to the corresponding wild type cells within the same plaques (Fig. 3b). Similarly, no differences were seen between knockout and wild type in *Il-1r*^*–/–*^ mixed chimeras (Suppl. Figure 1a–c). In *Nlrp3*^*–/–*^ mixed chimeras, however, NLRP3-deficient macrophages showed significantly reduced proliferation (6.26 ± 0.52% versus 8.17% ± 0.44% for WT macrophages). The rate of apoptosis did not differ between genotypes (Fig. 3b); meanwhile, WT macrophages showed greater relative accumulation in the plaque in contrast to the ratios of blood and aortic Ly6C^high^ monocytes (Fig. 3b). This observation, analogous to the phenotypes in *Cd36*^*–/–*^ and *Msr1*^*–/–*^ mixed chimeras, suggests that the proliferative advantage of *Nlrp3*^+*/*+^ plaque macrophages has led to a significant shift towards the wild type genotype within the same plaques (Fig. 3b). Although NLRP3, ASC, and Caspase-1 together form the inflammasome complex responsible for releasing mature IL-1β, NLRP3 appeared to promote plaque macrophage proliferation independently of the other inflammasome components.

**Fig. 4:**
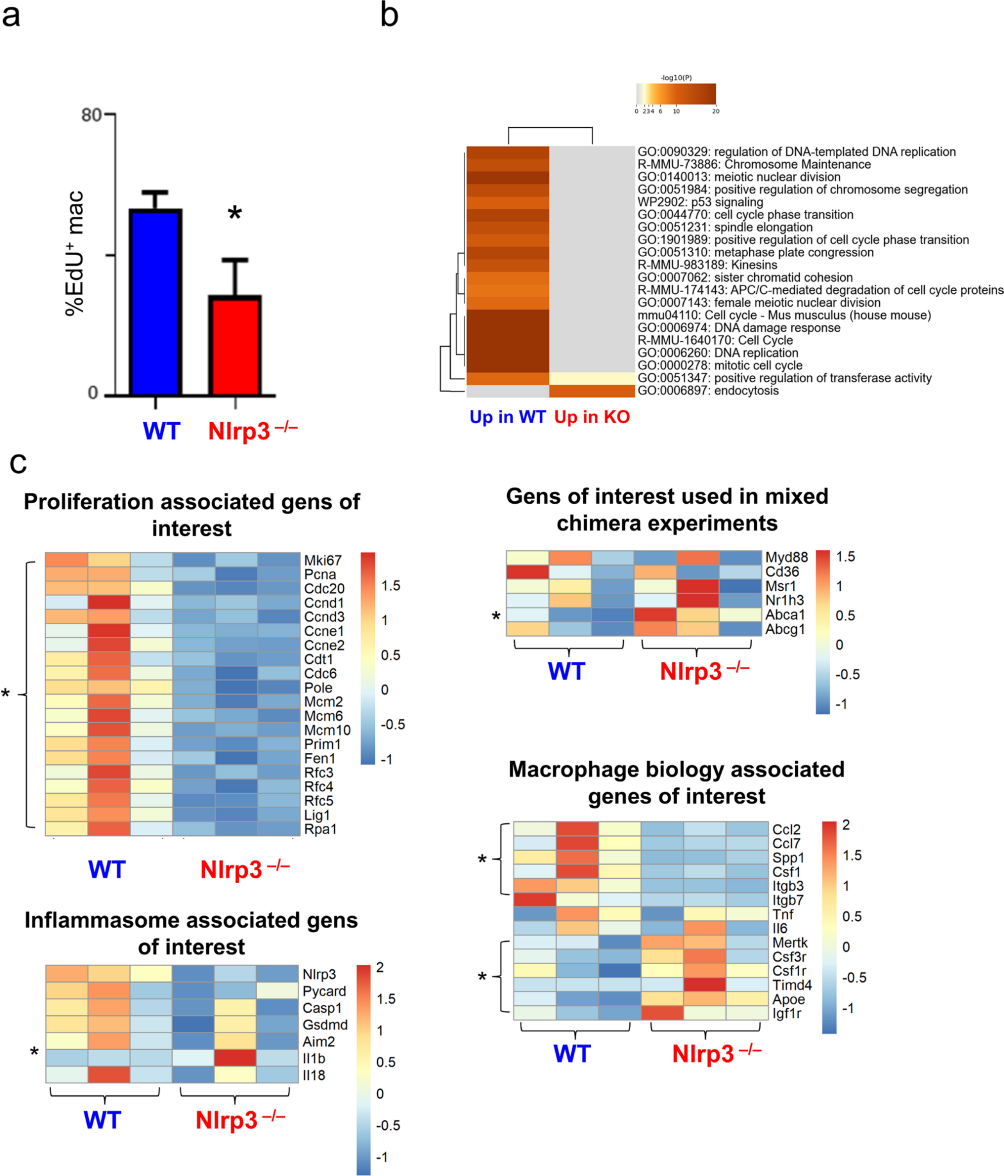
NLRP3 mediates oxLDL-stimulated bone marrow derived macrophage proliferation in vitro **a** BMDM were generated from WT and *Nlrp3*^−/−^ bone marrow cells and incubated with 20 µg/ml oxLDL and EdU for 12 h. Proliferation (EdU^+^) of BMDMs were analyzed by flow cytometry. Proliferation fraction is stated as mean ± SEM, n = 5 per group, *p < 0.05 denotes statistically significant differences, t-test. Bulk RNA-seq was performed of WT and *Nlrp3*^−/−^ BMDMs, n = 3, and analyzed in the following. **b** GO-Term Analysis of differentially regulated genes. **c** Heatmaps are shown for proliferation associated genes, inflammasome associated genes, genes previously used for mixed chimera experiments and macrophage genes of interest. Differentially regulated genes are marked with *, indicating p-adj. < 0.05

To validate our findings ex vivo, BMDM generated from WT and *Nlrp3*^*−/−*^ bone marrow cells were incubated with 20 µg/ml oxLDL and EdU for 12 h. EdU incorporation in BMDM was quantified by flow cytometry as a measure of macrophage proliferation (Fig. 4a). In line with our in vivo findings, oxLDL-stimulated *Nlrp3*^*−/−*^ macrophages proliferated less compared to *Nlrp3*^+*/*+^ macrophages (Fig. 4a).

We performed bulk RNA Sequencing to explore the effects on a transcriptomic level. 775 differentially expressed genes (DEG) were identified by DESeq2-Analysis. Mechanisms identified by GO Term Analysis in Metascape included upregulation of proliferatory components in wildtype BMDMs and upregulation of endocytosis in *Nlrp3*^*−/−*^ BMDMs (Fig. 4b). Proliferation associated genes such as *Mki67* and *Pcna* were downregulated in oxLDL-stimulated *Nlrp3*^*−/−*^ BMDMs. Except for (pro-)IL-1β, which was significantly upregulated in *Nlrp3*^*−/−*^ BMDM, other components of the NLRP3 inflammasome, IL-18, the alternative inflammasome component Absent in Melanoma 2 (*Aim2*) and Gasdermin (*Gsdmd*) were not differentially regulated in *Nlrp3*^*−/−*^ BMDM vs WT BMDM. Similarly, the other candidate genes involved in macrophage lipid import and export, which we had previously analyzed in the chimera experiments, were unaffected by *Nlrp3*-deficiency—except for an upregulation of the cholesterol exporter gene *Abca1* (Fig. 4c). Expression levels of efferocytosis receptor *Mertk* and growth factor receptors *Csf1r*, *Csf3r* and *Igf1r* were also elevated in *Nlrp3*^*−/−*^ BMDM compared to WT while levels of some chemokines and integrins were relatively reduced (Fig. 4c).

### Targeted inhibition of NLRP3 by MCC950 reduces the proliferation and accumulation of macrophages in mice

Exploring NLRP3 inhibition as a therapeutic strategy to target plaque macrophage proliferation beyond macrophage inflammation, we treated *Apoe*^*−/−*^ mice with the NLRP3 inhibitor MCC950. Macrophage proliferation and accumulation were quantified by flow cytometry and immunofluorescence histology.

**Fig. 5:**
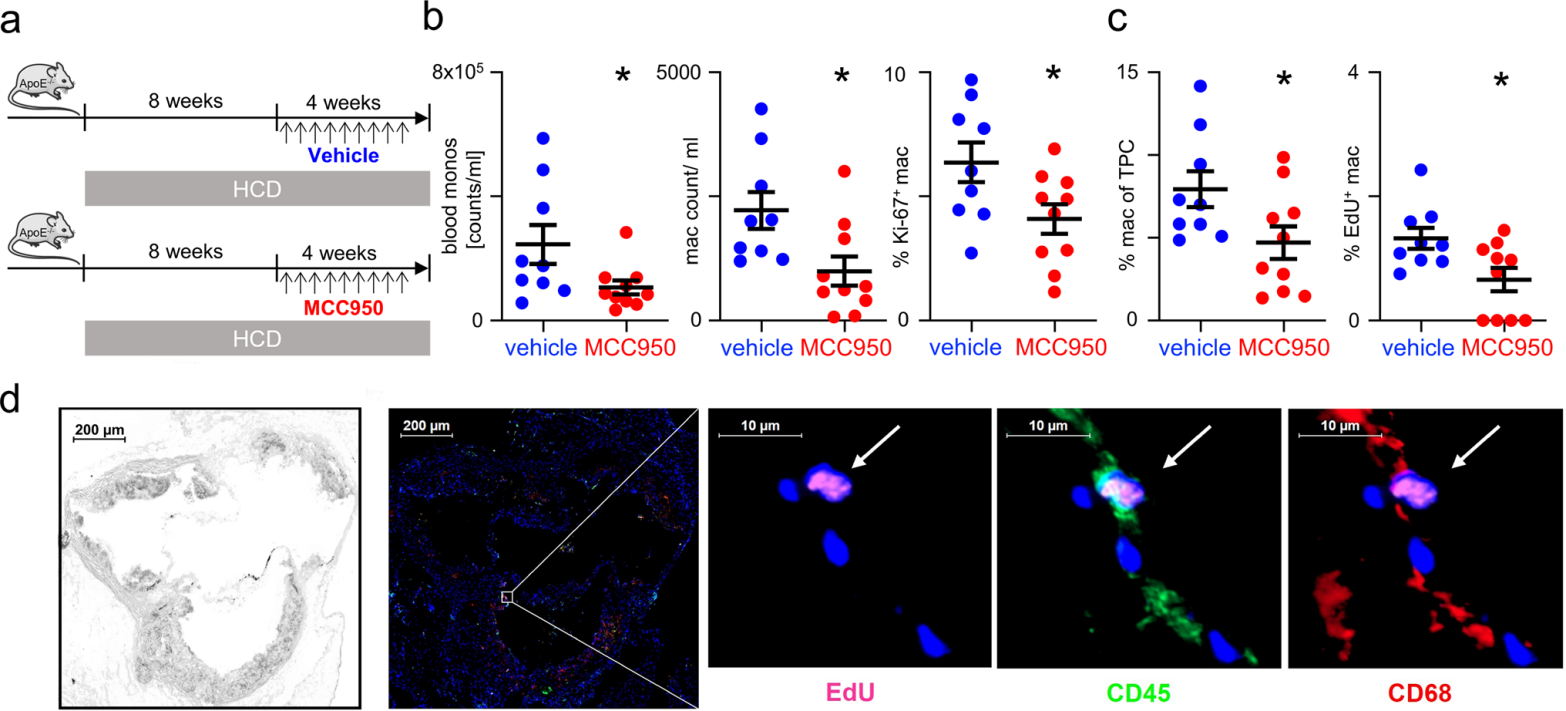
Pharmacological NLRP3 inhibition with MCC950 reduces the proliferation and accumulation of lesional macrophages in *Apoe*^*−/−*^ mice **a** *Apoe*^*−/−*^ mice were fed with a cholesterol diet (0.2% cholesterol) for 12 weeks. In the final 4 weeks, they were treated with either MCC950 (10 µg per g of body weight) or vehicle. **b** Blood monocytes, aortic macrophages, and proliferating aortic macrophages, as analyzed by flow cytometry, are shown as mean ± SEM; n = 9/10; *p < 0.05 indicates statistically significant differences as determined by unpaired t-test. **c** Macrophages (CD45^+^ and CD68^+^) among total plaque cells (TPC) and the number of proliferating macrophages were quantified in immunofluorescence images. Cells were manually counted in the immunofluorescence images of the aortic root to quantify the different subpopulations in the atherosclerotic lesion. Subpopulations are shown as mean ± SEM; n = 9/10; *p < 0.05 indicates statistically significant differences as determined by unpaired t-test. (d) Representative brightfield image and immunofluorescence (IF) image of an aortic root stained with DAPI (nuclei in blue), CD68 (macrophages in red), CD45 (leukocytes in green), and EdU (proliferating cells in pink). The representative enlargement of a plaque area shows different co-staining of a proliferating macrophage marked with a white arrow

*Apoe*^*−/−*^ mice were randomized into two groups, each comprising 5 male and 5 female mice, following 8 weeks of high-cholesterol diet feeding to establish atherosclerosis. The treatment group received MCC950 subcutaneously every other day for another 4 weeks while continuing the high-cholesterol diet, whereas control mice received vehicle treatment (Fig. 5a). Blood monocyte counts, aortic root macrophage counts, and macrophage proliferation (quantified with intracellular Ki-67 staining) were analyzed by flow cytometry (Fig. 5b). Complementarily, proliferative activity of CD45^+^ CD68^+^ plaque macrophages was quantified using immunofluorescent histology based on EdU incorporation (Fig. 5c).

In our model of established atherosclerosis (i.e., after 12 weeks on a high-cholesterol diet), 4 weeks of MCC950-induced NLRP3 inhibition led to a significant reduction in macrophage proliferation (Ki-67.^+^: 6.37 ± 0.8%, vs. 4.1 ± 0.59%, p = 0.03, and EdU: 3.3 ± 0.42%, vs. 1.65 ± 0.47%, p = 0.02) (Fig. 5b, c). Macrophages accumulation was also lower with MCC950 treatment (4437 ± 738 vs. 1990 ± 589 counts/ml by flow cytometry, and 7.93 ± 1.08% vs. 4.7 ± 0.98% by histology) (Fig. 5b, c). Plaque size, aortic macrophage cell death (as assessed by Caspase-3 staining), plasma cholesterol, body weight, and aorta weight were similar in MCC950- and vehicle-treated mice on the day of sacrifice (Suppl. Fig. S2b). Blood monocytes were significantly reduced in MCC950-treated mice versus vehicle (Fig. 5b) in line with previous reports [15]. Unlike previous studies, we initiated MCC950 treatment at a more advanced stage of atherosclerosis, when plaques were already established and local macrophage proliferation had become the primary driver of macrophage accumulation—surpassing monocyte infiltration and macrophage differentiation [12, 24, 31]

### MCC950 inhibits local macrophage proliferation independently of IL-1β in humans

**Fig. 6:**
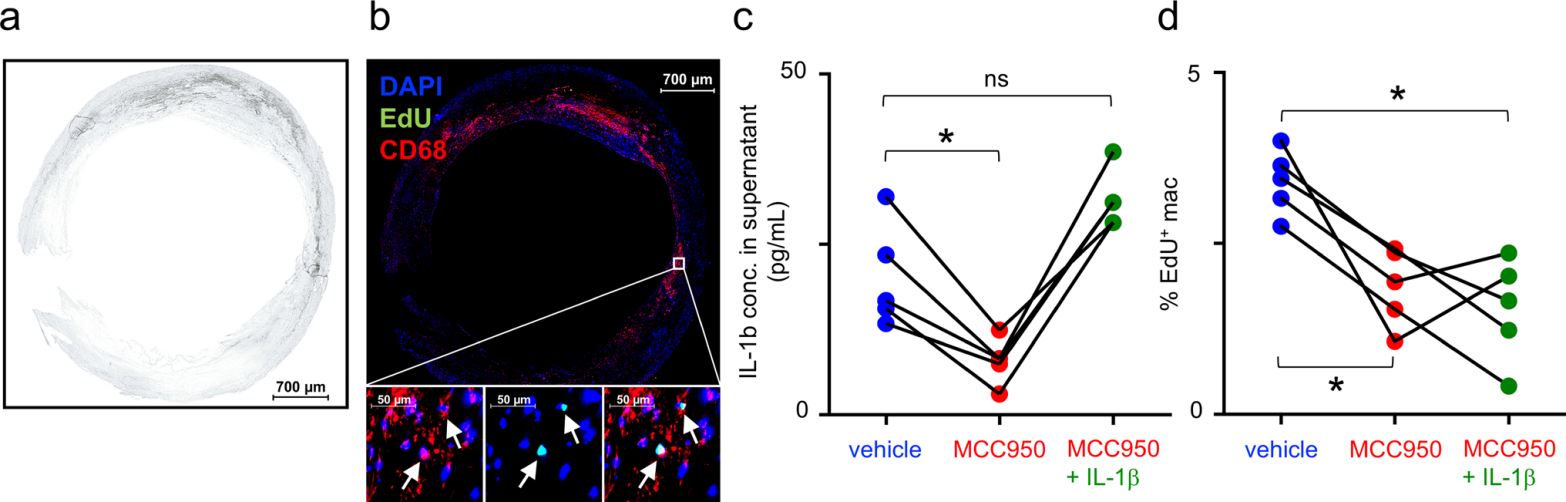
NLRP3 inhibition suppresses human macrophage proliferation independently of Interleukin-1β (IL-1β) **a** Representative brightfield and **b** immunofluorescence image of an atherosclerotic human carotid artery. Immunostaining shows nuclei (DAPI, blue), macrophages (CD68, red), and proliferating cells (EdU, green). The lower panel displays a magnified view of the boxed plaque region, presenting co-stained macrophages and proliferating cells, and their merged image. Proliferating macrophages (CD68 + /Edu + cells) are marked with white arrows. **c** IL-1β concentration in the supernatant of plaque section incubated for 24 h with vehicle, 1 µmol/L of MCC950 alone or with 125 pg/ml IL-1β. Each data set represents three sections of one human carotid plaque; n = 5; ns = not significant; *p < 0.05 indicates statistically significant differences as determined by one-way ANOVA. **d** Proportion of proliferating macrophages to total plaque-associated macrophages per section and condition. The respective cell populations were counted manually in immunofluorescence images; n = 5; ns = not significant; *p < 0.05 indicates statistically significant differences as determined one-way ANOVA

Finally, we tested whether NLRP3 inhibition by MCC950 also leads to reduced macrophage proliferation in humans. We collected carotid artery plaques from five patients and incubated three adjacent sections with EdU, vehicle or MCC950 ± IL-1β for 24 h. Macrophage proliferation was assessed by immunofluorescence staining for DAPI, anti-CD68, and EdU (Fig. 6).

MCC950 significantly reduced IL-1β levels in the supernatant of human carotid artery plaque sections. In a separate condition, IL-1β was supplemented alongside MCC950 (Fig. 6c). Macrophage proliferation decreased by more than 47% with MCC950 treatment and was not restored by the addition of IL-1β (Fig. 6d). These findings suggest that the inhibitory effect of MCC950 on macrophage proliferation is independent of IL-1β.

## Discussion

In this study, we aimed to determine the cellular processes that underlie lipid-triggered local macrophage proliferation in the atherosclerotic plaque and identify a possible therapeutic target for translational research in human atherosclerosis.

Knockout of the scavenger receptors *Cd36* and *Msr1* resulted in reduced lipid uptake and lower intracellular lipid content in macrophages, as well as a relative suppression of macrophage proliferation in both established and early atherosclerosis. Conversely, knockout of the cholesterol exporters *Abca1* and *Abcg1* increased macrophage proliferation in atherosclerosis, but also resulted in increased rates of apoptosis, suggesting that apoptosis dominates over proliferation of Mac-*Abc*-DKO macrophages (perhaps due to endoplasmic reticulum stress [9]), leading to a decrease in their number in advanced atherosclerosis. These results confirm our previous finding that decreased systemic cholesterol levels lead to reduced macrophage proliferation in the atherosclerotic plaque and are in agreement with similar results observed previously in murine hematopoietic stem cells, where *Abca1* and *Abcg1* depletion enhanced cell proliferation [9, 28, 29, 42].

In chimeric knockout models of *Cd36*, *Msr1*, or *Abca1* and *Abcg1*, monocyte and macrophage chimerisms in blood or aorta did not differ between wild type and knockout cells after 4 weeks of high-cholesterol diet, indicating that monocyte infiltration and macrophage differentiation in the nascent atherosclerotic plaque are unaffected by deletion of these genes. Conversely, after 12 weeks of high-cholesterol diet, we saw a predominance of wild type over knockout macrophages in the aorta, but not in the blood. Aortic wild type macrophages also showed higher rates of proliferation compared with *Msr1*^*−/−*^ and *Cd36*^*−/−*^ macrophages at both timepoints, whereas Mac-*Abc*-DKO showed higher rates of apoptosis when compared with wild type at the later timepoint. These results suggest that macrophage accumulation during plaque progression is due to a lipid-induced local proliferative effect, rather than increased infiltration.

We next investigated the intracellular processes by which cholesterol potentially drives local macrophage proliferation in the established plaque. Chimeras with knockouts of the cholesterol sensor *Lxrα/β* (which enhances gene expression of cholesterol exporters in response to increased cholesterol concentrations [21, 27, 37]) showed increased macrophage apoptosis and proliferation due to intracellular lipid accumulation. Chimeras with knockouts of *Myd88* (an adaptor protein involved in TLR signaling, macrophage oxLDL uptake, and monocyte recruitment to atherosclerotic lesions via downstream activation of NF-κB [35]) did not show any significant differences in chimerism, proliferation, or apoptosis when compared to the corresponding wild type cells. These results suggest that LXRα/β prevents macrophages from lipid overload induced apoptosis and proliferation.

As oxLDL and intracellularly accumulated cholesterol crystals are known to induce NLRP3 inflammasome activation [7, 19], we decided to investigate its role in macrophage proliferation. Deficiency of each of the three inflammasome proteins (NLRP3, ASC, Caspase 1) has been shown to reduce atherosclerotic lesion or plaque size [2, 7, 10, 22], and the inflammasome regulates the production and recruitment of monocytes through IL-1β in the early stages of atherosclerosis [15, 31, 35]. Surprisingly, only the absence of NLRP3 was associated with reductions in lesional macrophage proliferation and accumulation in our study. ASC and Caspase 1, components required to form the inflammasome, did not affect macrophage proliferation. This suggests that NLRP3 mediated lipid-driven macrophage proliferation in established atherosclerosis independent of the inflammasome complex. This is further supported by the observation that inflammasome-dependent production of IL-1β, which stimulates monocytopoiesis under atherogenic conditions [35], does not trigger plaque macrophage proliferation. Inflammasome-independent functions of NLRP3 are still ill-defined but have been observed before in other conditions [3, 16, 41]. This is the first report linking NLRP3 to plaque macrophage proliferation.

Translating these findings into a therapeutic approach, we treated *Apoe*^*−/−*^ mice with MCC950, an inhibitor targeting the NACHT domain of NLRP3, thereby preventing its conformational change and activation, and consequently blocking inflammasome assembly [5, 11, 18, 35]. NLRP3 is a promising therapeutic target; it can be activated by various stimuli, including intracellular cholesterol, and it regulates various proatherogenic processes [27, 30]. We initiated MCC950 treatment after atherosclerotic lesions had formed and plaque macrophages had accumulated. This is important because inflammasome inhibition by MCC950 in *Apoe*^*−/−*^ mice suppresses monocyte production and infiltration into the nascent plaque [15] (Fig. 5). However, we have previously demonstrated that after 8 weeks of high-cholesterol diet feeding in *Apoe*^*−/−*^ mice, macrophage accumulation is predominantly driven by local proliferation [12, 31]. Therefore, the reduced monocyte counts are insufficient to explain the lower macrophage numbers observed in advanced plaques of MCC950-treated mice, which are more likely due to suppressed local macrophage proliferation. Adding to the translational relevance of our findings, we demonstrated that MCC950-mediated NLRP3 inhibition reduced macrophage proliferation in human plaques ex vivo. We therefore consider NLRP3 a promising therapeutic target in atherosclerosis, as it combines classic inflammasome inhibition—reducing IL-1β and IL-18 release—with direct anti-proliferative effects on plaque macrophages. Notably, selective IL-1β blockade reduced major adverse cardiovascular events in patients post-acute coronary syndrome in the CANTOS trial at the expense of slightly more serious infections [41]. MCC950 was evaluated in a phase 1 clinical trial in rheumatoid arthritis but demonstrated hepatotoxicity in humans [13]. Currently, modern NLRP3 inhibitors, which are structurally related to MCC950 but with improved safety profiles, are undergoing clinical testing in neurologic and cardiometabolic diseases [31].

Taken together, we demonstrate that lipid accumulation in plaque macrophages promotes local proliferation—a process mediated by NLRP3 but independent of inflammasome activation. As a result, NLRP3 inhibition effectively slows macrophage proliferation and accumulation in atherosclerotic lesions in both mice and humans.

## Supplementary Information

Below is the link to the electronic supplementary material.

Supplementary file1 (DOCX 6463 kb)
