# Supplementary material for: NLRP3 mediates lipid-driven macrophage proliferation in established atherosclerosis
Source: Basic Res Cardiol. 2025 Sep 16;120(6):1141–54. doi: 10.1007/s00395-025-01137-6 (PMC12680701; doi:10.1007/s00395-025-01137-6)

**Supplemental Fig. 1** (a) For internal validation of the model, control chimeras were generated by lethally irradiating *Ldlr⁻/⁻* mice and reconstituting them with a 1:1 mixture of bone marrow from CD45.1⁺ WT and CD45.2⁺ WT donors. After 6 weeks of reconstitution, mice were fed a high-cholesterol diet (HCD) for 12 weeks. (b) Representative dot plots and gating strategy identifying Ly6C^high^ monocytes, macrophages, and their proliferating and apoptotic fraction. Cells originating from blood are gated in red; cells originating from aorta are gated in gray. Data are presented as mean ± SEM and individual proliferation and apoptotic fraction; n = 4 per group; ns = not significant as determined by paired t-test. (c) Intracellular Ki-67 and active Caspase-3 expression between WT and *Il-1r^−/−^* macrophages are presented as individual proliferation and apoptotic fraction; n = 6 per group; ns = non-significant differences between *Il-1r^+/+^* and *Il-1r^−/−^* as determined by paired t-test. Relative change in chimerism of blood monocytes, aortic monocytes, and aortic macrophages of *Il-1r^−/−^* chimera. The shift in chimerism is shown as mean ± SEM percentage change; n = 6 per group; ns = non-significant difference as determined by one-Way ANOVA.

**
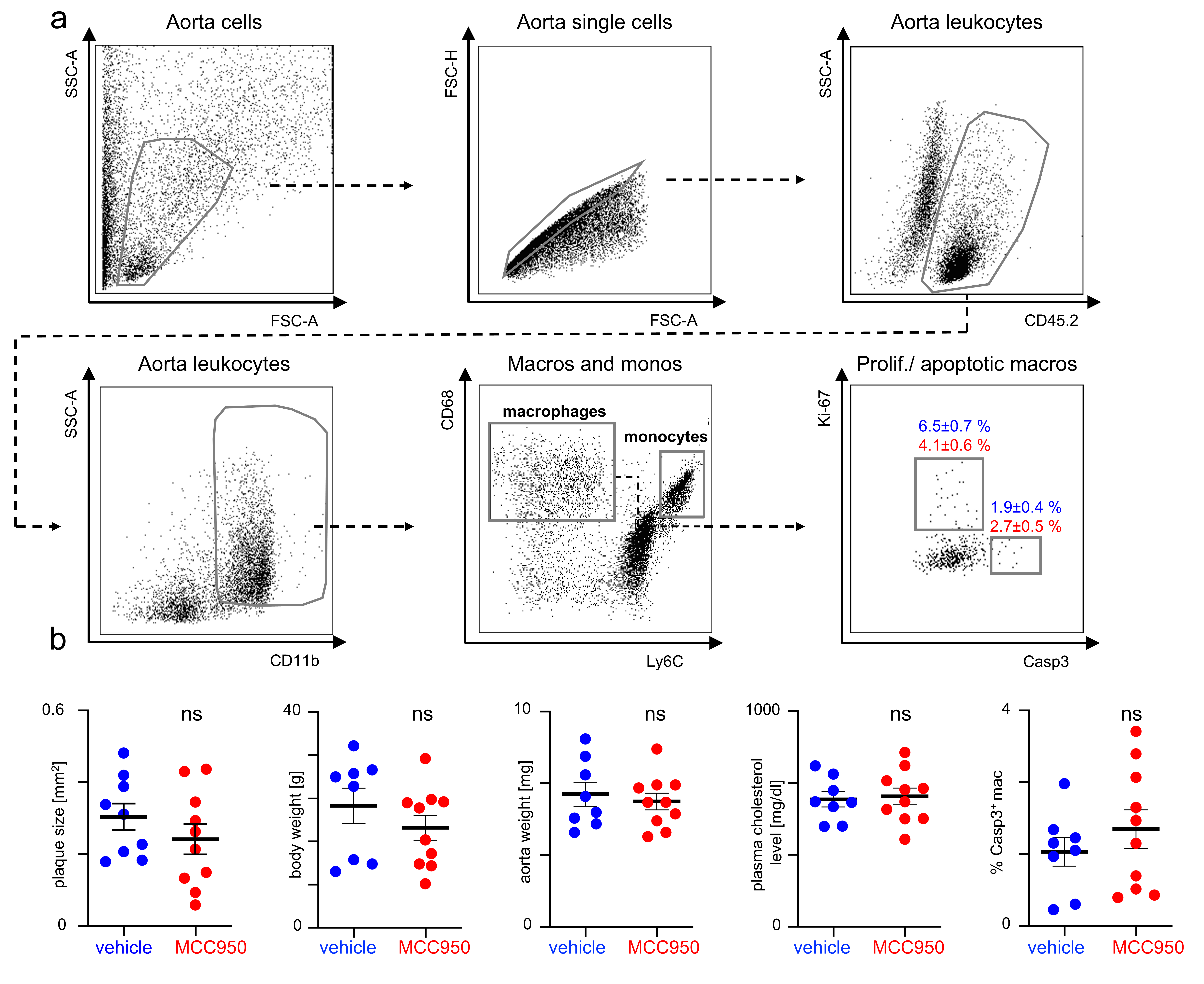
**

**Supplemental Fig. 2** (a) Representative gating strategy of *Apoe^−/−^* mice treated with or without MCC950 (10 µg per g body weight), identifying the fraction of proliferating and apoptotic macrophages in atherosclerotic aorta by flow cytometry. (b) Body weights, aorta weights, plasma cholesterol levels, and leukocyte numbers in the blood at the time of sacrifice in *Apoe^−/−^* mice with or without MCC950 treatment. Blood leukocytes were quantified by flow cytometry. Data are presented as mean ± SEM; n = 9/10; ns = not significant as determined by one-Way ANOVA.

**
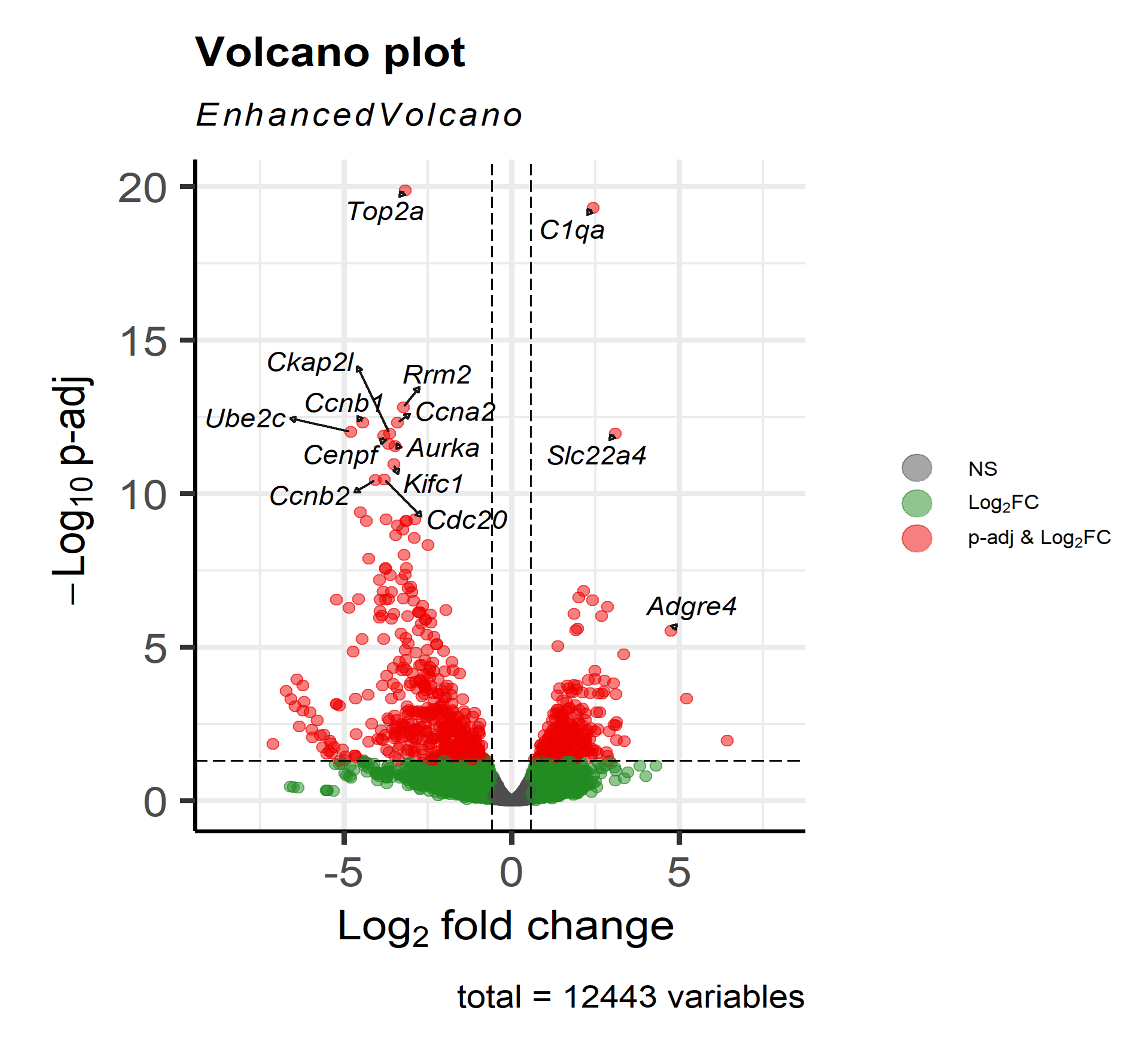
**

**Supplemental Fig. 3** (a) Bulk RNA-seq was performed of WT and *Nlrp3^-/-^* BMDMs, n=3, and the data are shown as a Volcano plot of differentially regulated genes. Wildtype is set as reference level.

**Supplemental Table 1** Antibodies used in flow cytometry


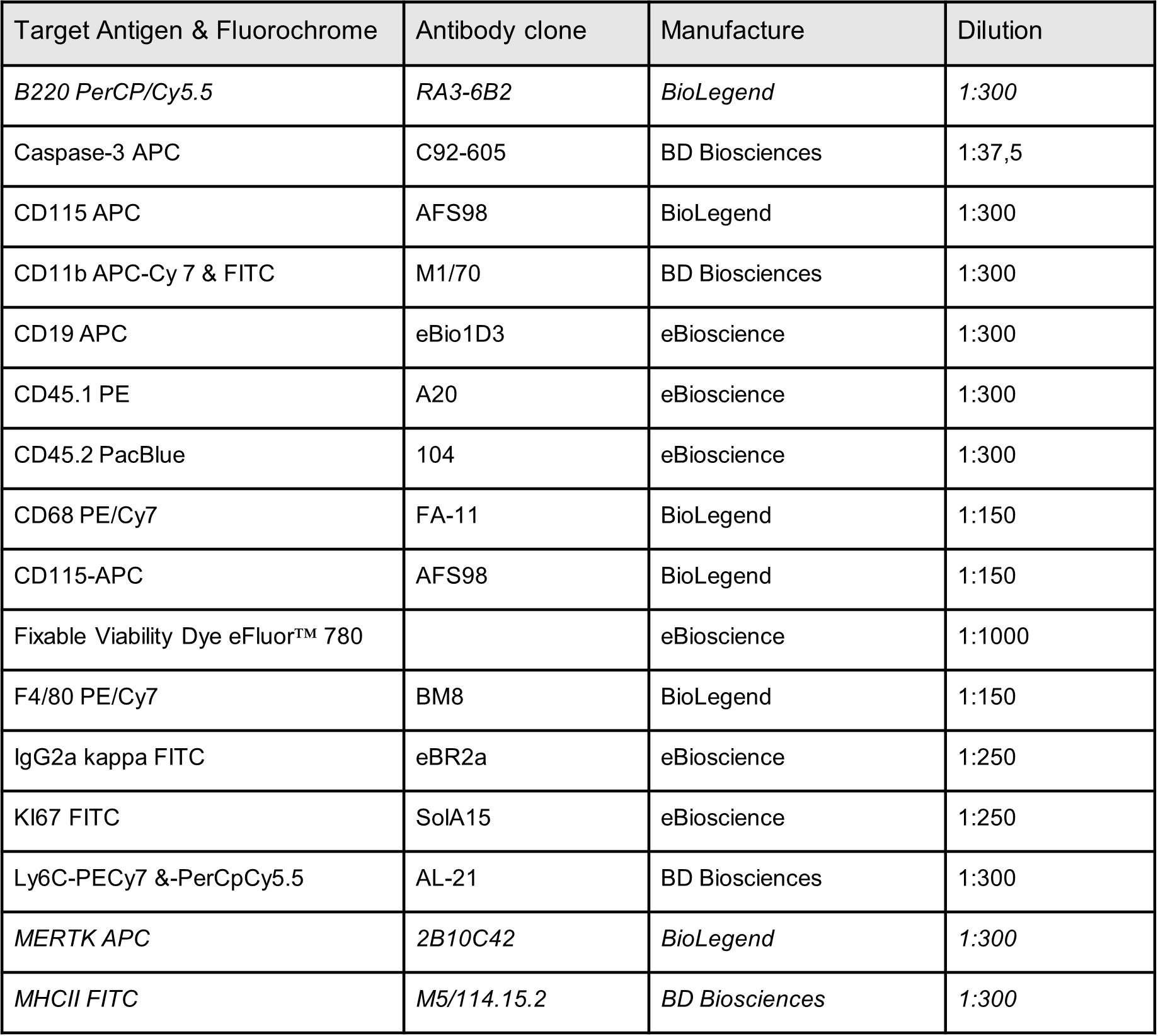


**Supplemental Table 2** Information of eCEA patients


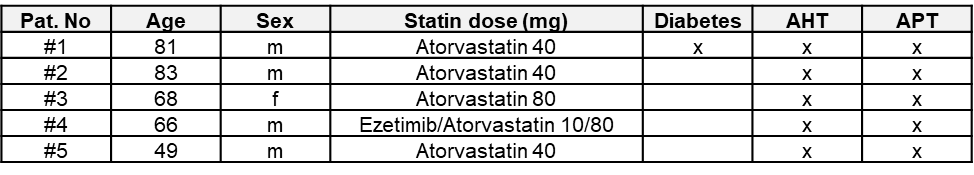

Supplement: Supplementary file 1 — Supplementary file1 (DOCX 6463 kb) [file 395_2025_1137_MOESM1_ESM.docx]
